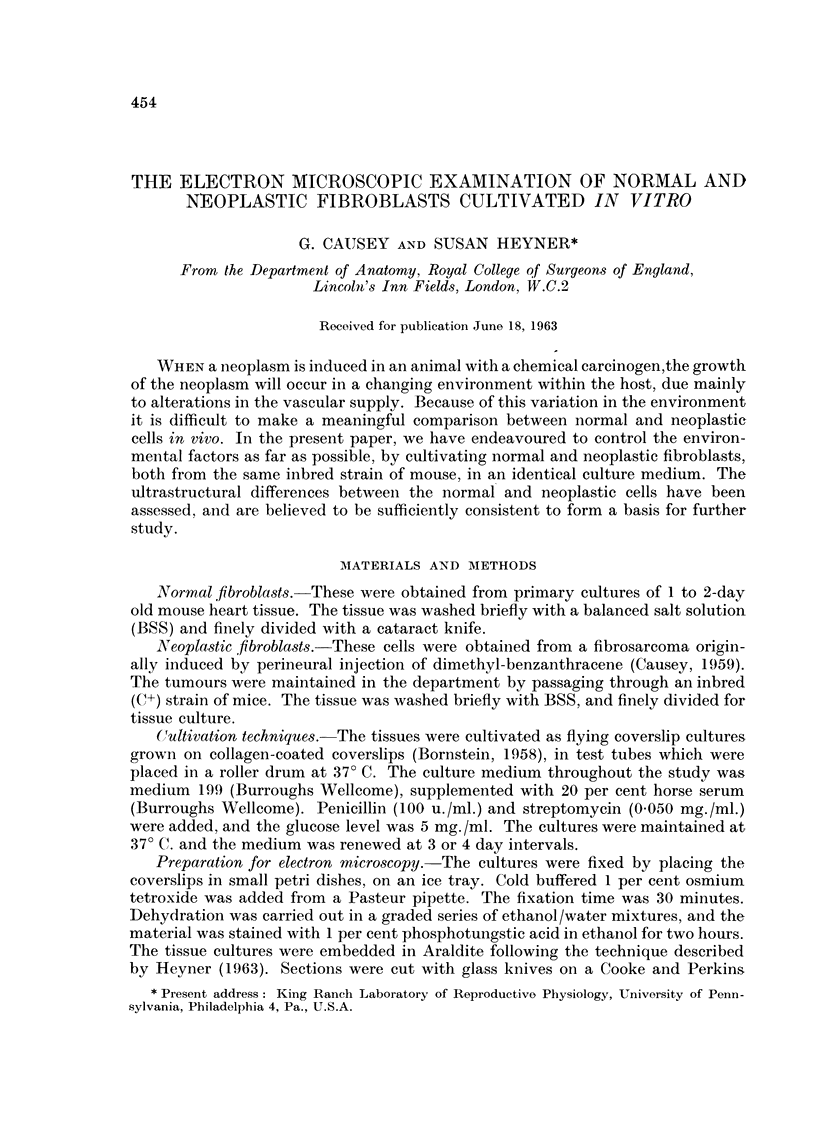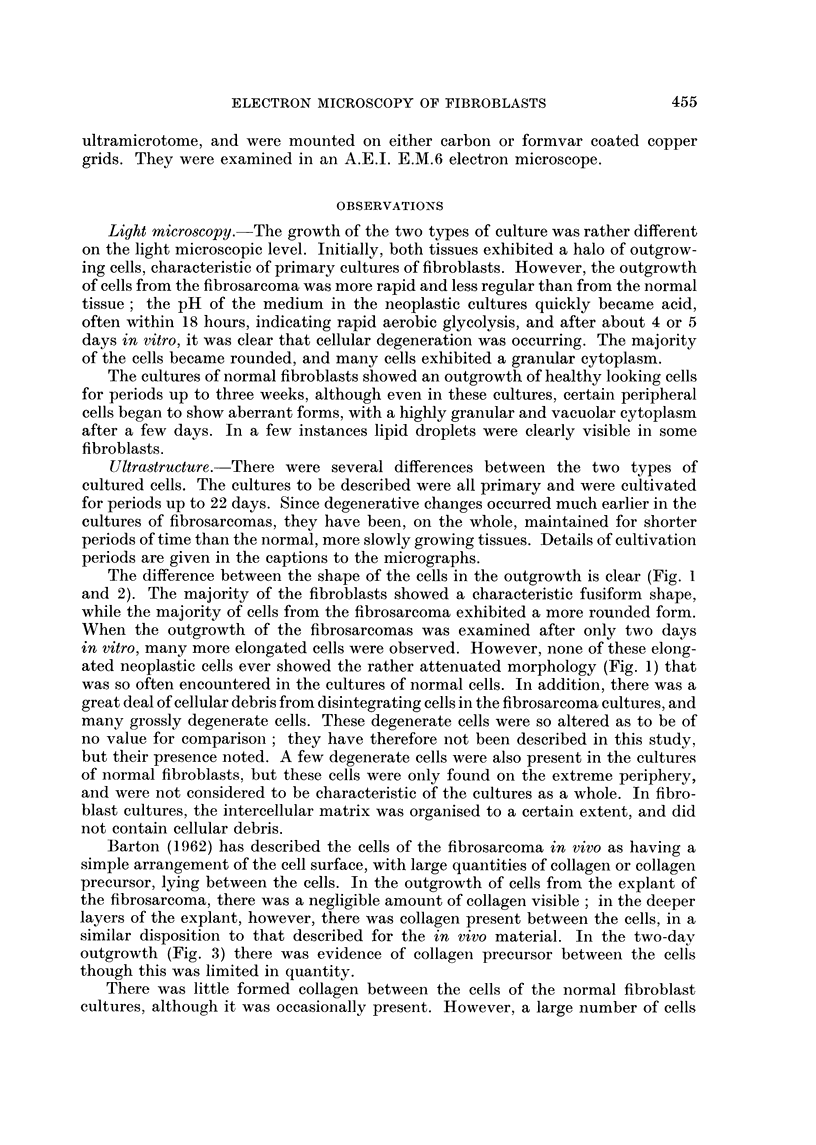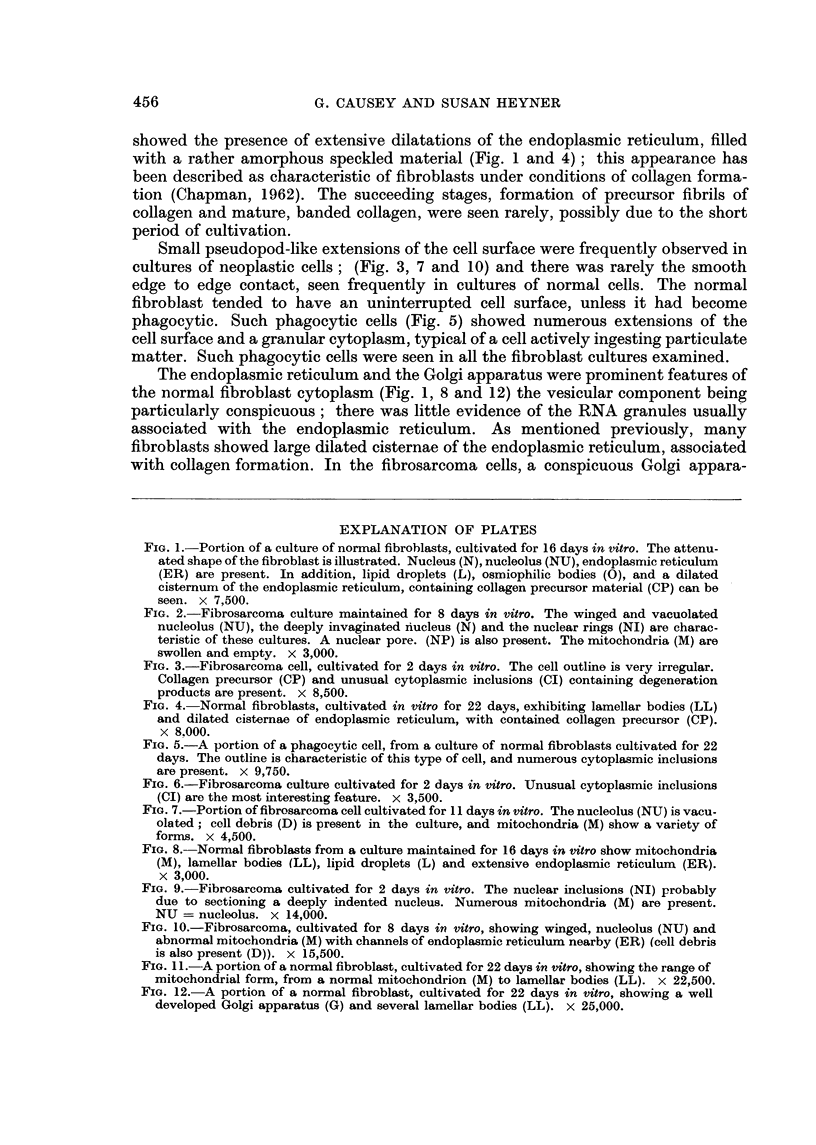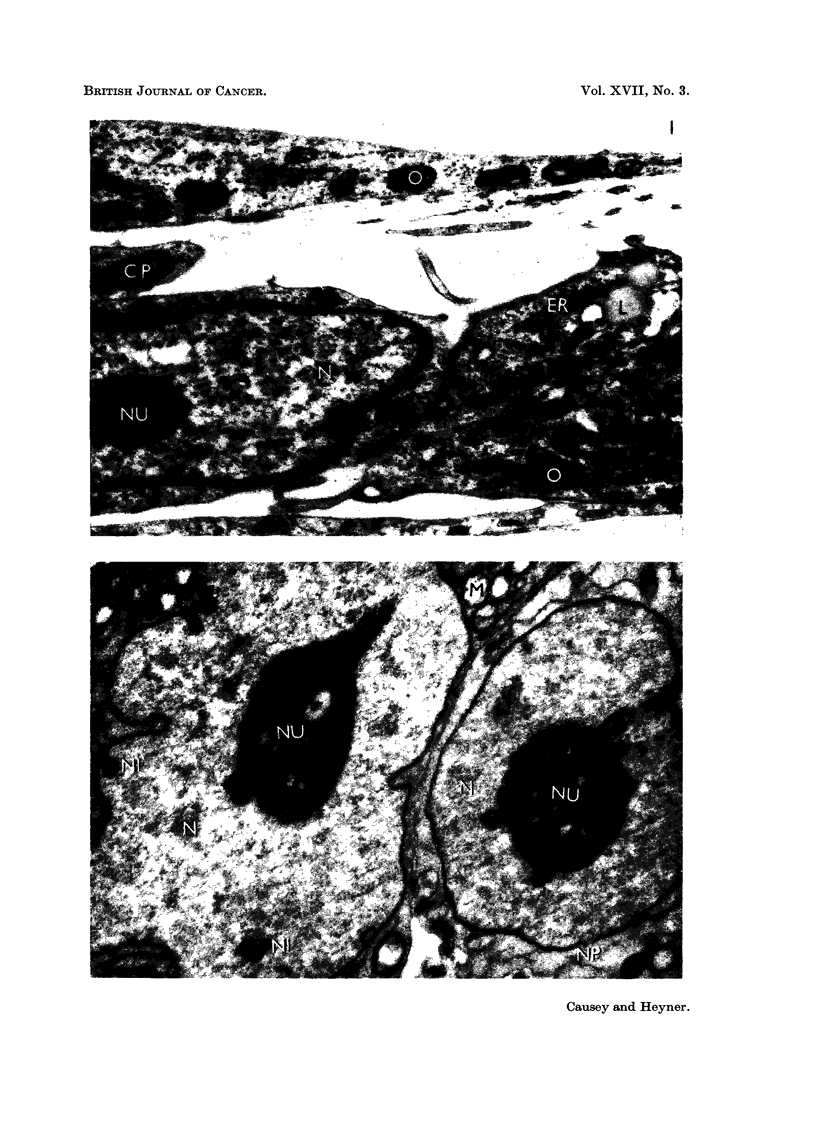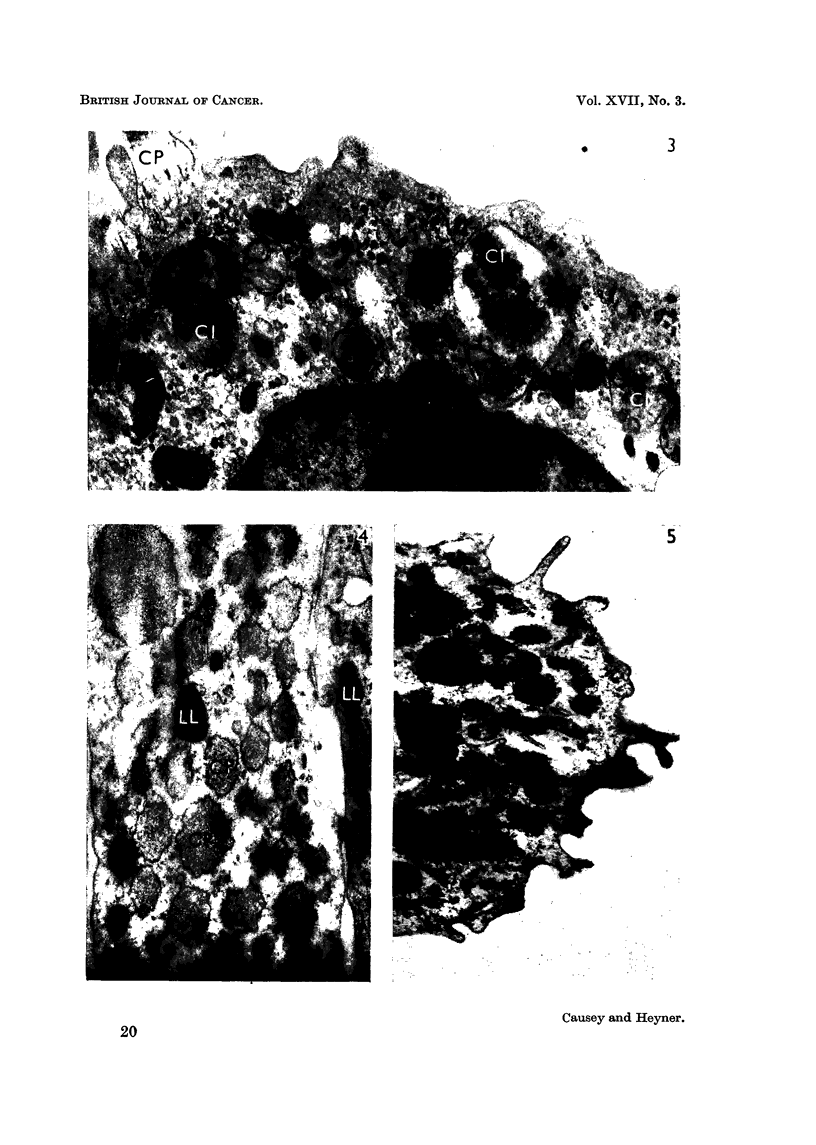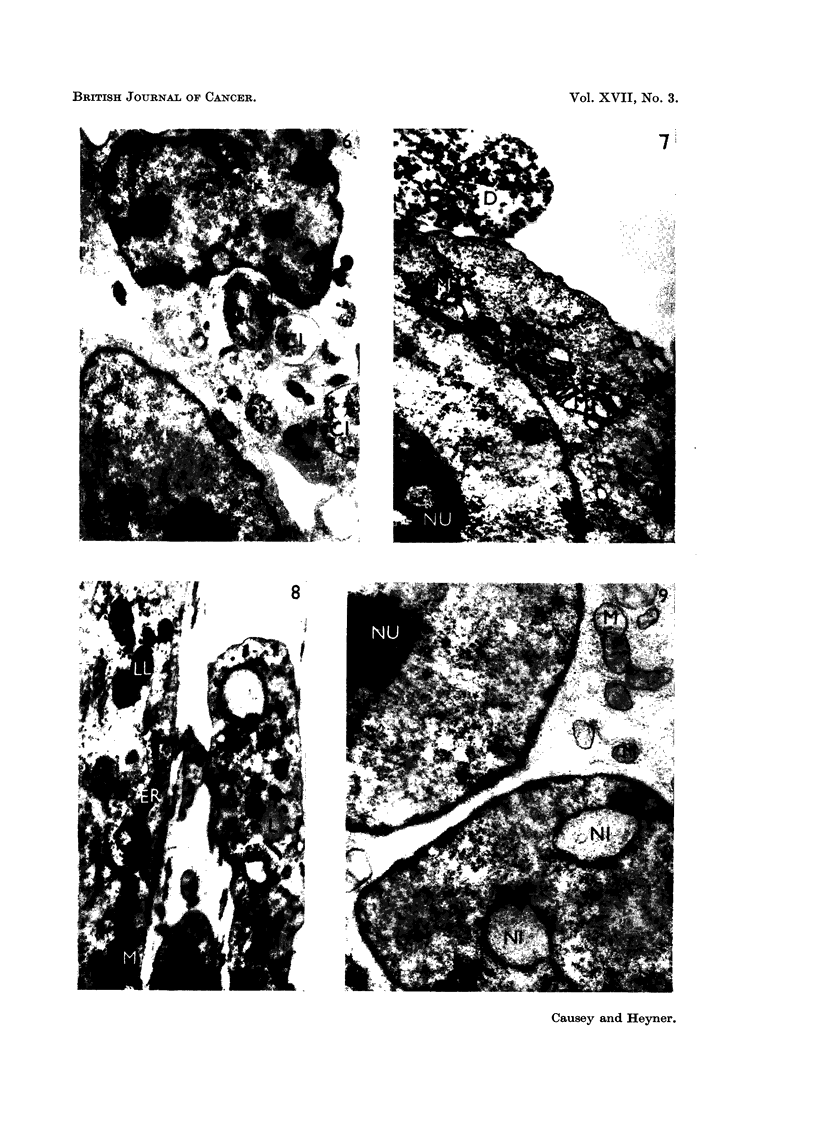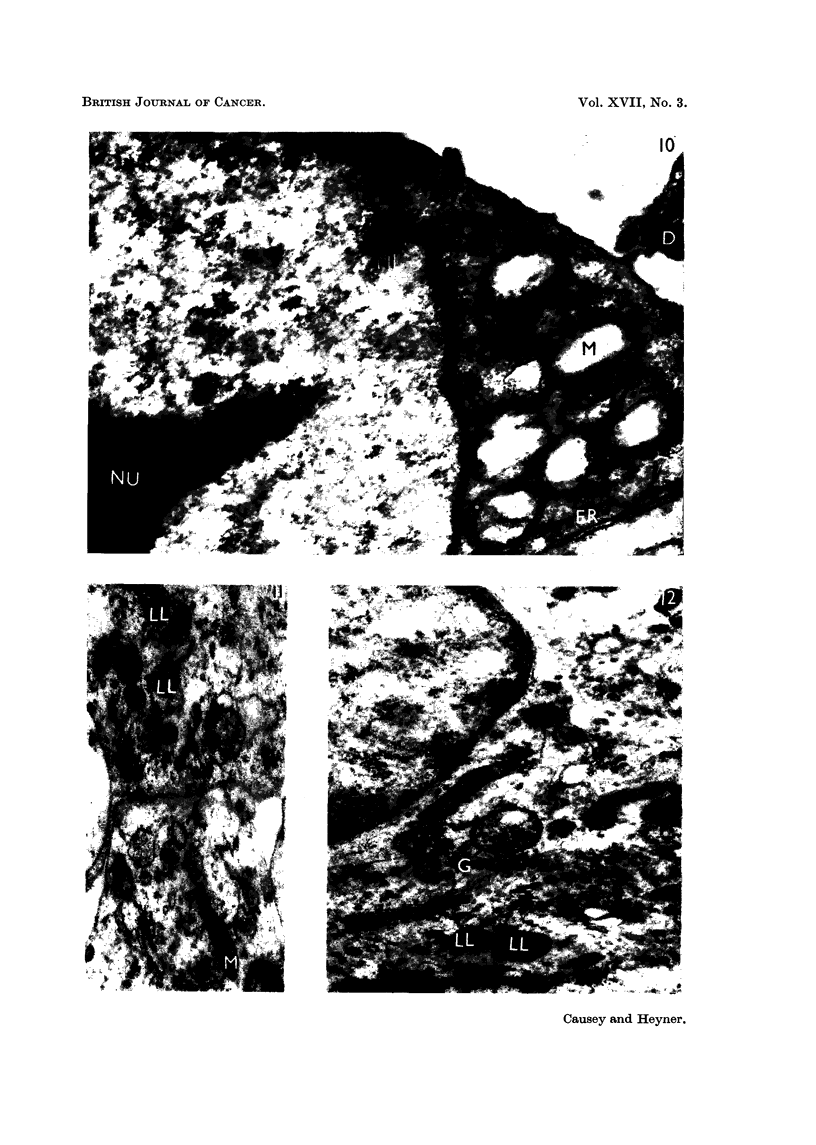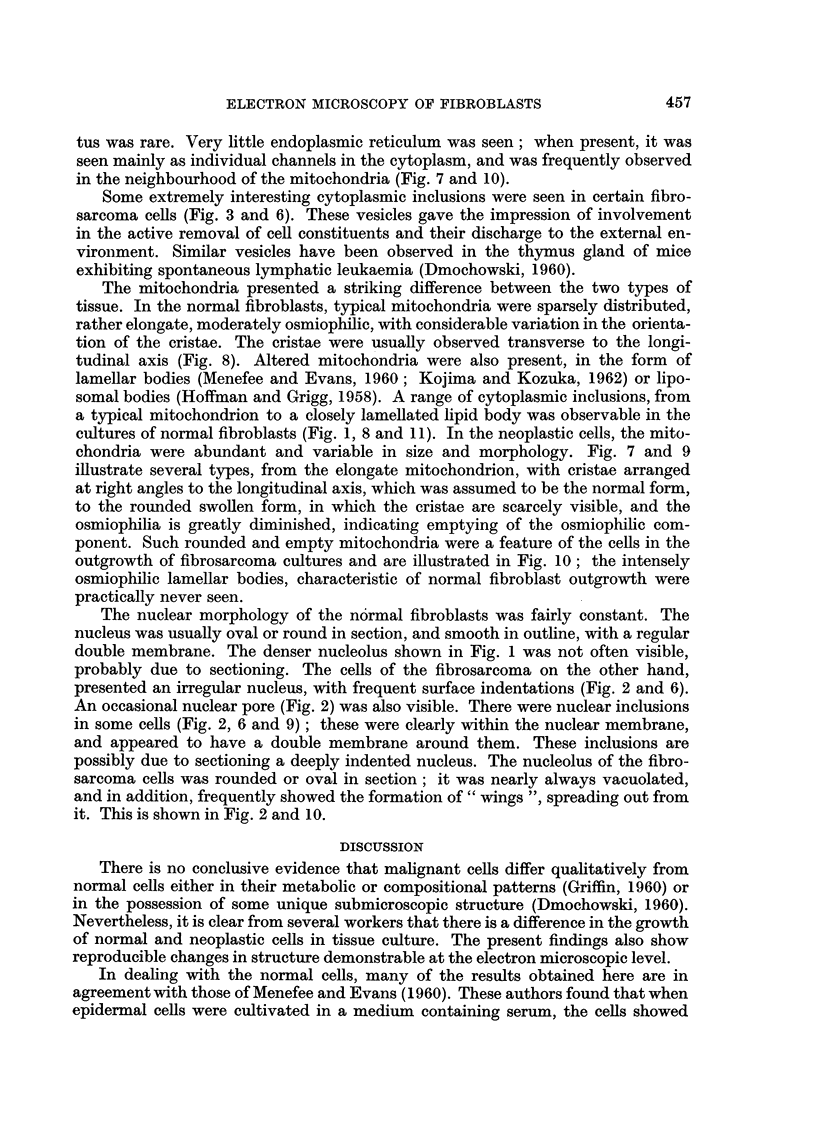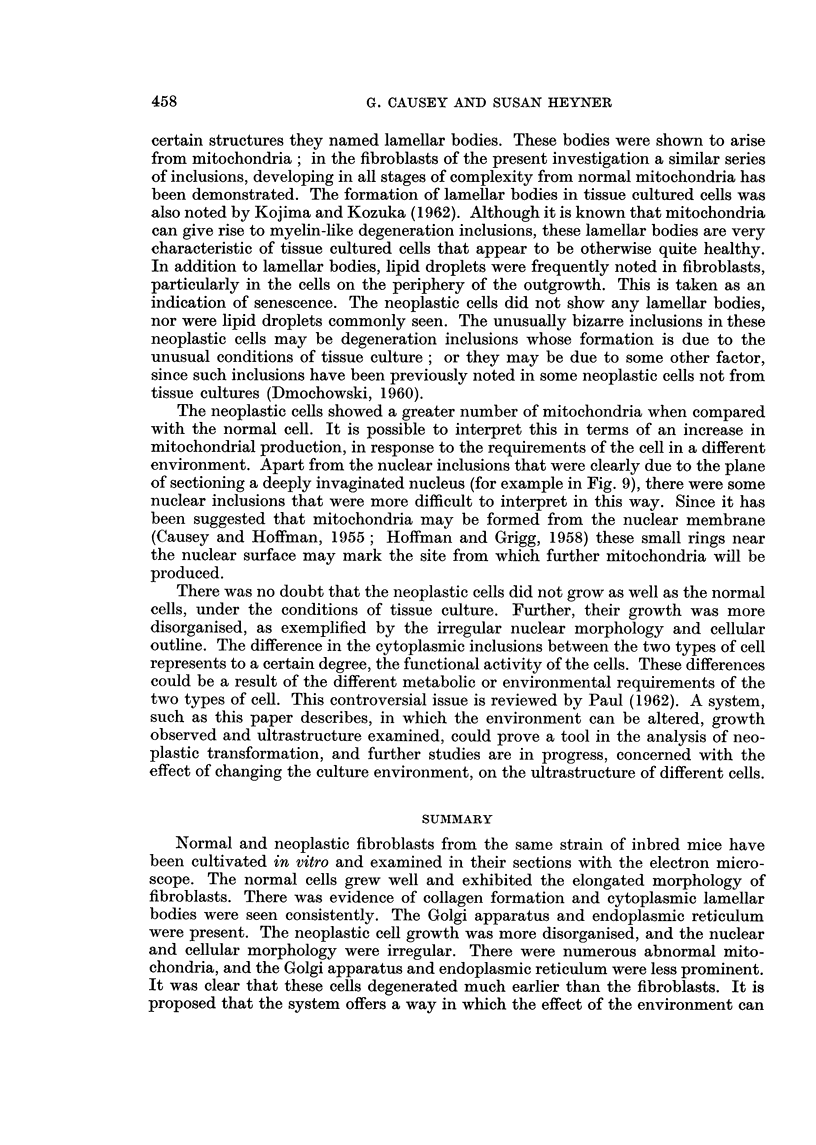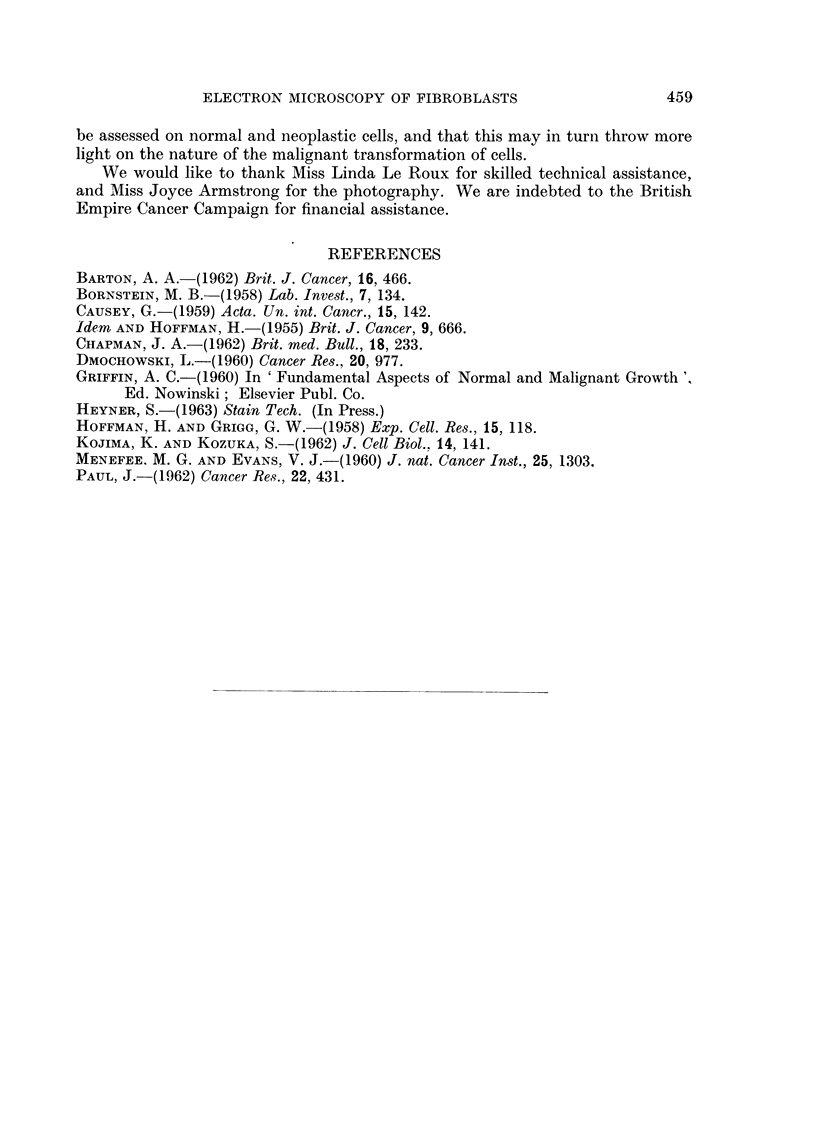# The Electron Microscopic Examination of Normal and Neoplastic Fibroblasts Cultivated In Vitro

**DOI:** 10.1038/bjc.1963.60

**Published:** 1963-09

**Authors:** G. Causey, Susan Heyner

## Abstract

**Images:**


### THE ELECTRON MICROSCOPIC EXAMINATION OF NORMAL AND NEOPLASTIC FIBROBLASTS CULTIVATED *IN VITRO*

G. CAUSEY AND SUSAN HEYNER*

*From the Department of Anatomy, Royal College of Surgeons of England, Lincoln's Inn Fields, London, W.C.2*

Received for publication June 18, 1963

WHEN a neoplasm is induced in an animal with a chemical carcinogen, the growth of the neoplasm will occur in a changing environment within the host, due mainly to alterations in the vascular supply. Because of this variation in the environment it is difficult to make a meaningful comparison between normal and neoplastic cells *in vivo*. In the present paper, we have endeavoured to control the environmental factors as far as possible, by cultivating normal and neoplastic fibroblasts, both from the same inbred strain of mouse, in an identical culture medium. The ultrastructural differences between the normal and neoplastic cells have been assessed, and are believed to be sufficiently consistent to form a basis for further study.

### MATERIALS AND METHODS

*Normal fibroblasts.*—These were obtained from primary cultures of 1 to 2-day old mouse heart tissue. The tissue was washed briefly with a balanced salt solution (BSS) and finely divided with a cataract knife.

*Neoplastic fibroblasts.*—These cells were obtained from a fibrosarcoma originally induced by perineural injection of dimethyl-benzanthracene (Causey, 1959). The tumours were maintained in the department by passaging through an inbred ($C^+$) strain of mice. The tissue was washed briefly with BSS, and finely divided for tissue culture.

*Cultivation techniques.*—The tissues were cultivated as flying coverslip cultures grown on collagen-coated coverslips (Bornstein, 1958), in test tubes which were placed in a roller drum at 37° C. The culture medium throughout the study was medium 199 (Burroughs Wellcome), supplemented with 20 per cent horse serum (Burroughs Wellcome). Penicillin (100 u./ml.) and streptomycin (0·050 mg./ml.) were added, and the glucose level was 5 mg./ml. The cultures were maintained at 37° C. and the medium was renewed at 3 or 4 day intervals.

*Preparation for electron microscopy.*—The cultures were fixed by placing the coverslips in small petri dishes, on an ice tray. Cold buffered 1 per cent osmium tetroxide was added from a Pasteur pipette. The fixation time was 30 minutes. Dehydration was carried out in a graded series of ethanol/water mixtures, and the material was stained with 1 per cent phosphotungstic acid in ethanol for two hours. The tissue cultures were embedded in Araldite following the technique described by Heyner (1963). Sections were cut with glass knives on a Cooke and Perkins

* Present address: King Ranch Laboratory of Reproductive Physiology, University of Pennsylvania, Philadelphia 4, Pa., U.S.A.

ultramicrotome, and were mounted on either carbon or formvar coated copper grids. They were examined in an A.E.I. E.M.6 electron microscope.

### OBSERVATIONS

*Light microscopy.*—The growth of the two types of culture was rather different on the light microscopic level. Initially, both tissues exhibited a halo of outgrowing cells, characteristic of primary cultures of fibroblasts. However, the outgrowth of cells from the fibrosarcoma was more rapid and less regular than from the normal tissue ; the pH of the medium in the neoplastic cultures quickly became acid, often within 18 hours, indicating rapid aerobic glycolysis, and after about 4 or 5 days *in vitro*, it was clear that cellular degeneration was occurring. The majority of the cells became rounded, and many cells exhibited a granular cytoplasm.

The cultures of normal fibroblasts showed an outgrowth of healthy looking cells for periods up to three weeks, although even in these cultures, certain peripheral cells began to show aberrant forms, with a highly granular and vacuolar cytoplasm after a few days. In a few instances lipid droplets were clearly visible in some fibroblasts.

*Ultrastructure.*—There were several differences between the two types of cultured cells. The cultures to be described were all primary and were cultivated for periods up to 22 days. Since degenerative changes occurred much earlier in the cultures of fibrosarcomas, they have been, on the whole, maintained for shorter periods of time than the normal, more slowly growing tissues. Details of cultivation periods are given in the captions to the micrographs.

The difference between the shape of the cells in the outgrowth is clear (Fig. 1 and 2). The majority of the fibroblasts showed a characteristic fusiform shape, while the majority of cells from the fibrosarcoma exhibited a more rounded form. When the outgrowth of the fibrosarcomas was examined after only two days *in vitro*, many more elongated cells were observed. However, none of these elongated neoplastic cells ever showed the rather attenuated morphology (Fig. 1) that was so often encountered in the cultures of normal cells. In addition, there was a great deal of cellular debris from disintegrating cells in the fibrosarcoma cultures, and many grossly degenerate cells. These degenerate cells were so altered as to be of no value for comparison ; they have therefore not been described in this study, but their presence noted. A few degenerate cells were also present in the cultures of normal fibroblasts, but these cells were only found on the extreme periphery, and were not considered to be characteristic of the cultures as a whole. In fibroblast cultures, the intercellular matrix was organised to a certain extent, and did not contain cellular debris.

Barton (1962) has described the cells of the fibrosarcoma *in vivo* as having a simple arrangement of the cell surface, with large quantities of collagen or collagen precursor, lying between the cells. In the outgrowth of cells from the explant of the fibrosarcoma, there was a negligible amount of collagen visible ; in the deeper layers of the explant, however, there was collagen present between the cells, in a similar disposition to that described for the *in vivo* material. In the two-day outgrowth (Fig. 3) there was evidence of collagen precursor between the cells though this was limited in quantity.

There was little formed collagen between the cells of the normal fibroblast cultures, although it was occasionally present. However, a large number of cells showed the presence of extensive dilatations of the endoplasmic reticulum, filled with a rather amorphous speckled material (Fig. 1 and 4); this appearance has been described as characteristic of fibroblasts under conditions of collagen formation (Chapman, 1962). The succeeding stages, formation of precursor fibrils of collagen and mature, banded collagen, were seen rarely, possibly due to the short period of cultivation.

Small pseudopod-like extensions of the cell surface were frequently observed in cultures of neoplastic cells; (Fig. 3, 7 and 10) and there was rarely the smooth edge to edge contact, seen frequently in cultures of normal cells. The normal fibroblast tended to have an uninterrupted cell surface, unless it had become phagocytic. Such phagocytic cells (Fig. 5) showed numerous extensions of the cell surface and a granular cytoplasm, typical of a cell actively ingesting particulate matter. Such phagocytic cells were seen in all the fibroblast cultures examined.

The endoplasmic reticulum and the Golgi apparatus were prominent features of the normal fibroblast cytoplasm (Fig. 1, 8 and 12) the vesicular component being particularly conspicuous; there was little evidence of the RNA granules usually associated with the endoplasmic reticulum. As mentioned previously, many fibroblasts showed large dilated cisternae of the endoplasmic reticulum, associated with collagen formation. In the fibrosarcoma cells, a conspicuous Golgi appara-

---

EXPLANATION OF PLATES

FIG. 1.—Portion of a culture of normal fibroblasts, cultivated for 16 days *in vitro*. The attenuated shape of the fibroblast is illustrated. Nucleus (N), nucleolus (NU), endoplasmic reticulum (ER) are present. In addition, lipid droplets (L), osmiophilic bodies (O), and a dilated cisternum of the endoplasmic reticulum, containing collagen precursor material (CP) can be seen. × 7,500.

FIG. 2.—Fibrosarcoma culture maintained for 8 days *in vitro*. The winged and vacuolated nucleolus (NU), the deeply invaginated nucleus (N) and the nuclear rings (NI) are characteristic of these cultures. A nuclear pore. (NP) is also present. The mitochondria (M) are swollen and empty. × 3,000.

FIG. 3.—Fibrosarcoma cell, cultivated for 2 days *in vitro*. The cell outline is very irregular. Collagen precursor (CP) and unusual cytoplasmic inclusions (CI) containing degeneration products are present. × 8,500.

FIG. 4.—Normal fibroblasts, cultivated *in vitro* for 22 days, exhibiting lamellar bodies (LL) and dilated cisternae of endoplasmic reticulum, with contained collagen precursor (CP). × 8,000.

FIG. 5.—A portion of a phagocytic cell, from a culture of normal fibroblasts cultivated for 22 days. The outline is characteristic of this type of cell, and numerous cytoplasmic inclusions are present. × 9,750.

FIG. 6.—Fibrosarcoma culture cultivated for 2 days *in vitro*. Unusual cytoplasmic inclusions (CI) are the most interesting feature. × 3,500.

FIG. 7.—Portion of fibrosarcoma cell cultivated for 11 days *in vitro*. The nucleolus (NU) is vacuolated; cell debris (D) is present in the culture, and mitochondria (M) show a variety of forms. × 4,500.

FIG. 8.—Normal fibroblasts from a culture maintained for 16 days *in vitro* show mitochondria (M), lamellar bodies (LL), lipid droplets (L) and extensive endoplasmic reticulum (ER). × 3,000.

FIG. 9.—Fibrosarcoma cultivated for 2 days *in vitro*. The nuclear inclusions (NI) probably due to sectioning a deeply indented nucleus. Numerous mitochondria (M) are present. NU = nucleolus. × 14,000.

FIG. 10.—Fibrosarcoma, cultivated for 8 days *in vitro*, showing winged, nucleolus (NU) and abnormal mitochondria (M) with channels of endoplasmic reticulum nearby (ER) (cell debris is also present (D)). × 15,500.

FIG. 11.—A portion of a normal fibroblast, cultivated for 22 days *in vitro*, showing the range of mitochondrial form, from a normal mitochondrion (M) to lamellar bodies (LL). × 22,500.

FIG. 12.—A portion of a normal fibroblast, cultivated for 22 days *in vitro*, showing a well developed Golgi apparatus (G) and several lamellar bodies (LL). × 25,000.

Causey and Heyner.

Causey and Heyner.

Causey and Heyner.

Causey and Heyner.

tus was rare. Very little endoplasmic reticulum was seen ; when present, it was seen mainly as individual channels in the cytoplasm, and was frequently observed in the neighbourhood of the mitochondria (Fig. 7 and 10).

Some extremely interesting cytoplasmic inclusions were seen in certain fibrosarcoma cells (Fig. 3 and 6). These vesicles gave the impression of involvement in the active removal of cell constituents and their discharge to the external environment. Similar vesicles have been observed in the thymus gland of mice exhibiting spontaneous lymphatic leukaemia (Dmochowski, 1960).

The mitochondria presented a striking difference between the two types of tissue. In the normal fibroblasts, typical mitochondria were sparsely distributed, rather elongate, moderately osmiophilic, with considerable variation in the orientation of the cristae. The cristae were usually observed transverse to the longitudinal axis (Fig. 8). Altered mitochondria were also present, in the form of lamellar bodies (Menefee and Evans, 1960 ; Kojima and Kozuka, 1962) or liposomal bodies (Hoffman and Grigg, 1958). A range of cytoplasmic inclusions, from a typical mitochondrion to a closely lamellated lipid body was observable in the cultures of normal fibroblasts (Fig. 1, 8 and 11). In the neoplastic cells, the mitochondria were abundant and variable in size and morphology. Fig. 7 and 9 illustrate several types, from the elongate mitochondrion, with cristae arranged at right angles to the longitudinal axis, which was assumed to be the normal form, to the rounded swollen form, in which the cristae are scarcely visible, and the osmiophilia is greatly diminished, indicating emptying of the osmiophilic component. Such rounded and empty mitochondria were a feature of the cells in the outgrowth of fibrosarcoma cultures and are illustrated in Fig. 10 ; the intensely osmiophilic lamellar bodies, characteristic of normal fibroblast outgrowth were practically never seen.

The nuclear morphology of the normal fibroblasts was fairly constant. The nucleus was usually oval or round in section, and smooth in outline, with a regular double membrane. The denser nucleolus shown in Fig. 1 was not often visible, probably due to sectioning. The cells of the fibrosarcoma on the other hand, presented an irregular nucleus, with frequent surface indentations (Fig. 2 and 6). An occasional nuclear pore (Fig. 2) was also visible. There were nuclear inclusions in some cells (Fig. 2, 6 and 9) ; these were clearly within the nuclear membrane, and appeared to have a double membrane around them. These inclusions are possibly due to sectioning a deeply indented nucleus. The nucleolus of the fibrosarcoma cells was rounded or oval in section ; it was nearly always vacuolated, and in addition, frequently showed the formation of " wings ", spreading out from it. This is shown in Fig. 2 and 10.

### DISCUSSION

There is no conclusive evidence that malignant cells differ qualitatively from normal cells either in their metabolic or compositional patterns (Griffin, 1960) or in the possession of some unique submicroscopic structure (Dmochowski, 1960). Nevertheless, it is clear from several workers that there is a difference in the growth of normal and neoplastic cells in tissue culture. The present findings also show reproducible changes in structure demonstrable at the electron microscopic level.

In dealing with the normal cells, many of the results obtained here are in agreement with those of Menefee and Evans (1960). These authors found that when epidermal cells were cultivated in a medium containing serum, the cells showed certain structures they named lamellar bodies. These bodies were shown to arise from mitochondria ; in the fibroblasts of the present investigation a similar series of inclusions, developing in all stages of complexity from normal mitochondria has been demonstrated. The formation of lamellar bodies in tissue cultured cells was also noted by Kojima and Kozuka (1962). Although it is known that mitochondria can give rise to myelin-like degeneration inclusions, these lamellar bodies are very characteristic of tissue cultured cells that appear to be otherwise quite healthy. In addition to lamellar bodies, lipid droplets were frequently noted in fibroblasts, particularly in the cells on the periphery of the outgrowth. This is taken as an indication of senescence. The neoplastic cells did not show any lamellar bodies, nor were lipid droplets commonly seen. The unusually bizarre inclusions in these neoplastic cells may be degeneration inclusions whose formation is due to the unusual conditions of tissue culture ; or they may be due to some other factor, since such inclusions have been previously noted in some neoplastic cells not from tissue cultures (Dmochowski, 1960).

The neoplastic cells showed a greater number of mitochondria when compared with the normal cell. It is possible to interpret this in terms of an increase in mitochondrial production, in response to the requirements of the cell in a different environment. Apart from the nuclear inclusions that were clearly due to the plane of sectioning a deeply invaginated nucleus (for example in Fig. 9), there were some nuclear inclusions that were more difficult to interpret in this way. Since it has been suggested that mitochondria may be formed from the nuclear membrane (Causey and Hoffman, 1955 ; Hoffman and Grigg, 1958) these small rings near the nuclear surface may mark the site from which further mitochondria will be produced.

There was no doubt that the neoplastic cells did not grow as well as the normal cells, under the conditions of tissue culture. Further, their growth was more disorganised, as exemplified by the irregular nuclear morphology and cellular outline. The difference in the cytoplasmic inclusions between the two types of cell represents to a certain degree, the functional activity of the cells. These differences could be a result of the different metabolic or environmental requirements of the two types of cell. This controversial issue is reviewed by Paul (1962). A system, such as this paper describes, in which the environment can be altered, growth observed and ultrastructure examined, could prove a tool in the analysis of neoplastic transformation, and further studies are in progress, concerned with the effect of changing the culture environment, on the ultrastructure of different cells.

### SUMMARY

Normal and neoplastic fibroblasts from the same strain of inbred mice have been cultivated *in vitro* and examined in their sections with the electron microscope. The normal cells grew well and exhibited the elongated morphology of fibroblasts. There was evidence of collagen formation and cytoplasmic lamellar bodies were seen consistently. The Golgi apparatus and endoplasmic reticulum were present. The neoplastic cell growth was more disorganised, and the nuclear and cellular morphology were irregular. There were numerous abnormal mitochondria, and the Golgi apparatus and endoplasmic reticulum were less prominent. It was clear that these cells degenerated much earlier than the fibroblasts. It is proposed that the system offers a way in which the effect of the environment can be assessed on normal and neoplastic cells, and that this may in turn throw more light on the nature of the malignant transformation of cells.

We would like to thank Miss Linda Le Roux for skilled technical assistance, and Miss Joyce Armstrong for the photography. We are indebted to the British Empire Cancer Campaign for financial assistance.

### REFERENCES

BARTON, A. A.—(1962) *Brit. J. Cancer*, **16**, 466.
BORNSTEIN, M. B.—(1958) *Lab. Invest.*, **7**, 134.
CAUSEY, G.—(1959) *Acta. Un. int. Cancr.*, **15**, 142.
*Idem* AND HOFFMAN, H.—(1955) *Brit. J. Cancer*, **9**, 666.
CHAPMAN, J. A.—(1962) *Brit. med. Bull.*, **18**, 233.
DMOCHOWSKI, L.—(1960) *Cancer Res.*, **20**, 977.
GRIFFIN, A. C.—(1960) In ' Fundamental Aspects of Normal and Malignant Growth ', Ed. Nowinski ; Elsevier Publ. Co.
HEYNER, S.—(1963) *Stain Tech.* (In Press.)
HOFFMAN, H. AND GRIGG, G. W.—(1958) *Exp. Cell. Res.*, **15**, 118.
KOJIMA, K. AND KOZUKA, S.—(1962) *J. Cell Biol.*, **14**, 141.
MENEFEE. M. G. AND EVANS, V. J.—(1960) *J. nat. Cancer Inst.*, **25**, 1303.
PAUL, J.—(1962) *Cancer Res.*, **22**, 431.